# Spatiotemporal Dynamics of Covert vs. Overt Emotional Face Processing in Dysphoria

**DOI:** 10.3389/fnbeh.2022.920989

**Published:** 2022-07-05

**Authors:** Fern Jaspers-Fayer, Antonio Maffei, Jennifer Goertzen, Killian Kleffner, Ambra Coccaro, Paola Sessa, Mario Liotti

**Affiliations:** ^1^Laboratory for Affective and Developmental Neuroscience, Department of Psychology, Simon Fraser University, Burnaby, BC, Canada; ^2^Department of Developmental and Social Psychology, University of Padua, Padua, Italy; ^3^Padova Neuroscience Center, University of Padua, Padua, Italy

**Keywords:** ERPs, dysphoria, emotion, attention, faces, N170, depression

## Abstract

People at risk of developing clinical depression exhibit attentional biases for emotional faces. To clarify whether such effects occur at an early, automatic, or at a late, deliberate processing stage of emotional processing, the present study used high-density electroencephalography during both covert and overt processing of sad, fearful, happy, and neutral expressions in healthy participants with high dysphoria (*n* = 16) and with low dysphoria (*n* = 19). A state-of-the-art non-parametric permutation-based statistical approach was then used to explore the effects of emotion, attentional task demands, and group. Behaviorally, participants responded faster and more accurately when overtly categorizing happy faces and they were slower and less accurate when categorizing sad and fearful faces, independent of the dysphoria group. Electrophysiologically, in an early time-window (N170: 140–180 ms), there was a significant main effect for the dysphoria group, with greater negative voltage for the high vs. low dysphoria group over the left-sided temporo-occipital scalp. Furthermore, there was a significant group by emotional interaction, with the high dysphoria group displaying greater negative amplitude N170 for happy than fearful faces. Attentional task demands did not influence such early effects. In contrast, in an intermediate time-window (EPN: 200–400 ms) and in a late time-window (LPP: 500–750 ms) there were no significant main effects nor interactions involving the dysphoria Group. The LPP results paralleled the behavioral results, with greater LPP voltages for sad and fearful relative to happy faces only in the overt task, but similarly so in the two dysphoria groups. This study provides novel evidence that alterations in face processing in dysphoric individuals can be seen at the early stages of face perception, as indexed by the N170, although not in the form of a typical pattern of mood-congruent attentional bias. In contrast, intermediate (EPN) and late (LPP) stages of emotional face processing appear unaffected by dysphoria. Importantly, the early dysphoria effect appears to be independent of the top-down allocation of attention, further supporting the idea that dysphoria may influence a stage of automatic emotional appraisal. It is proposed that it may be a consequence of a shift from holistic to feature-based processing of facial expressions, or may be due to the influence of negative schemas acting as a negative context for emotional facial processing.

## Introduction

Human facial expressions are the main non-verbal channel for socio-emotional communication, and as such efficient and accurate emotional face processing is crucial for social functioning. Facial expressions simultaneously transmit and evoke an emotion (Wild et al., 2001) and attentional bias mechanisms may impact their salience and interpretation in people with depressive syndromes, impairing social functioning and perpetuating distress (Segrin and Abramson, 1994). People at risk of developing clinical depression may show these attentional biases for emotional faces in the early phases of their disease course, before the onset of clinical illness.

### Cognitive Bias in Depression

Early cognitive vulnerability models of affective disorders posited that adverse early-life events cause an individual to develop negative associations (“schemas”) that bias attention toward negative stimuli in the environment and additionally bias the interpretation of these stimuli as more negative, increasing their salience in the future and creating persistent negative mood. This cycle may begin before and ultimately precipitate clinical illness onset (Teasdale, 1988; Beevers and Carver, 2003; Beck, 2008). Such models have prompted a plethora of studies on symptom-congruent attention biases, most of which have used behavioral reaction time (RT) outcome measures. The consensus among early studies was that people with anxiety were more likely to exhibit robust early, automatic attentional capture by symptom-congruent (i.e., anxiety-related) stimuli, while people with depression were more likely to exhibit impaired attentional disengagement from symptom-congruent (i.e., depression-related) stimuli (see Bradley et al., 1997; Mogg and Bradley, 1998, 2005).

Informed by such studies, Beevers (2005) dual-process model of depression proposed that at an early stage of disease, *depression vulnerability* may be characterized by dysfunctional associative networks, with increased salience to negative stimuli and increased individual risk for a depressive episode. In contrast, a failure of slower inhibition mechanisms would set in at a later stage, during the full-blown clinical depression. As a result, mood-congruent attentional biases at a behavioral level have been explored in individuals with dysphoria, a condition in which individuals report elevated depressive symptoms on a psychometric instrument designed to assess them, but where these individuals have not formally been diagnosed with major depressive disorder or dysthymia (but may be susceptible to develop clinical depression, Kendall et al., 1987; Frewen and Dozois, 2005). In fact, biased attention to negative stimuli has been reported in individuals with sub-clinical depressive symptoms, as well as in individuals undergoing sad mood induction. Therefore, an attentional bias toward negative stimuli might be associated with sub-clinical depression, rather than being a marker of clinical depression *per se* (for a meta-analysis, see Peckham et al., 2010).

Reaction time measures, however, cannot easily tease apart different processing stages, since they may combine multiple effects in the one RT outcome measure. The high temporal resolution of event-related potentials (ERPs) can help delineate different processing stages more precisely, but to date, limited attempts have been made to explore the neural correlates of emotional information processing in participants with either subclinical depression (i.e., dysphoria) or diagnosed clinical depression.

### Emotional Face Processing and Event-Related Potentials

In the healthy adult literature, three ERP components have consistently been associated with processing emotional faces: the N170, the Early Posterior Negativity (EPN), and the Late Positive Potential (LPP). Reviewing all of these components may help elucidate different information processing stages in dysphoria.

The face-sensitive N170 component (approximately 140–180 ms) is recorded over the temporo-occipital scalp sites and is thought to reflect the early encoding of face structure and configuration (Bentin and Deouell, 2000; Rossion and Jacques, 2012). The strongest and most replicated emotion-related modulation of the N170 is an enhancement of fearful compared to neutral faces (Batty and Taylor, 2003; Hinojosa et al., 2015; Schindler and Bublatsky, 2020), but similar N170 modulations have also been reported for other emotions (happy, angry, and sad) relative to neutral faces (Hinojosa et al., 2015; Schindler and Bublatsky, 2020; Maffei et al., 2021).

Notably, some studies, employing a linked mastoid reference, have found the N170 emotion-related modulation to be absent, being replaced by an early positive emotion modulation (120–180 ms) over the frontocentral scalp, with greater amplitudes in response to emotional, particularly fearful, compared to neutral faces (Eimer and Holmes, 2002; Williams, 2006; Eimer et al., 2008; discussed by Maffei et al., 2021).

A second well-established ERP marker of emotion processing is the EPN (∼180–350 ms), a negative-going amplitude deflection distributed over the parieto-occipital scalp, typically obtained by calculating a difference wave between emotional and neutral stimuli. In healthy adults, the EPN is thought to reflect enhanced processing of emotionally salient faces in general, with a particular sensitivity for threatening faces (Schupp et al., 2003, 2004).

The third robust emotion-related ERP modulation is the enhancement of the LPP, a sustained wave (from approximately 400 ms to over 1 s, depending on stimuli exposure time) broadly distributed over the posterior scalp. LPP modulation is thought to reflect the increased allocation of processing/working memory resources to the motivational relevance of emotional stimuli (Schupp et al., 2006; Hajcak et al., 2010). As a result, the LPP can be attenuated by top-down regulation strategies, such as suppression and reappraisal (Hajcak et al., 2010).

### Event-Related Potential Emotion Face Processing in Depression

Most early ERP studies in clinical depression have focused specifically on the amplitude and/or latency of the P300 (Picton, 1992), an ERP component related to the allocation of attentional resources to non-emotional infrequent targets (oddballs) during stimulus evaluation and target detection (e.g., Liotti and Mayberg, 2001). Although some studies have manipulated the emotional valence of the stimuli (e.g., Cavanagh and Geisler, 2006; Krompinger and Simons, 2009), they have rarely reported on non-P300 components.

Several more recent studies in depression have focused on tasks with emotionally valenced stimuli, like emotional faces. Results have been mixed. Smaller N170 voltage amplitudes have been reported in clinical depression, independent of expression, relative to healthy controls (Dai and Feng, 2012; Chen et al., 2014) with the smallest voltage amplitudes in recurrent-depression patients (Chen et al., 2014). However, other studies have found no significant N170 voltage amplitude differences (Foti et al., 2010; Jaworska et al., 2012). An early negativity bias in clinical depression has been found only in some studies, in the form of higher N170 voltage amplitudes for sad faces relative to happy and neutral faces for depressed participants (Chen et al., 2014; Zhao et al., 2015; Dai et al., 2016). Evidence for a lack of a late positivity bias present in normal aging has been reported in old-age depression, with LPP amplitudes in elderly controls being larger for happy relative to sad faces, while no differences were present in the depressed group (Zhou et al., 2018).

Of the studies that have looked at ERP components in response to emotional faces in clinical depression, most have used covert tasks, where emotion was task-irrelevant. This is because covert tasks are thought to best activate early, automatic attentional capture by symptom-congruent stimuli. Very few EEG face studies have employed overt tasks (Dai et al., 2016; Zhou et al., 2018). Overt evaluation of emotional stimuli in clinical should be further explored since, in clinical depression, we might be just as interested in the attentional disengagement from symptom-congruent stimuli (Yiend, 2010; Epp et al., 2012).

### Event-Related Potential Emotion Face Processing in Subclinical Depression

A number of ERP face studies have explored the impact of subclinical depression or dysphoria on emotional processing. Bistricky et al. (2014) recorded EEG in an overt emotional oddball task in which participants responded to an infrequently presented target emotion and inhibited responses to an infrequently presented distracter emotion. They compared a dysphoric group with past clinical depression with a dysphoric group without prior depression history and a never-depressed non-dysphoric group. They found greater P3 amplitude to sad faces relative to happy faces only in dysphoric participants with past depression, while participants with dysphoria without a depression history did *not* display such attentional bias to sad faces. No differences among groups were found for the preceding frontocentral N2 to emotional distracters (Bistricky et al., 2014).

Buodo et al. (2015) recorded EEG during a covert task involving perceptual discrimination with task-irrelevant sad, happy, and neutral faces and employed an average mastoid reference. There were no significant N170 amplitude differences as a function of emotion or group, but the frontal P200 was larger for sad than neutral facial expressions; this pattern, however, did not vary significantly between dysphoric and control groups.

Dai et al. (2016) recorded EEG in an overt task of valence rating of happy, sad, and neutral faces. For the subclinical depression group, there were no N170 amplitude differences as a function of emotion or group, but for a later posterior P2 (150–320 ms)- coinciding with the EPN- there was greater amplitude for happy faces compared to a non-dysphoric control group (Dai et al., 2016).

Xu et al. (2018) employed magnetoencephalography (MEG) in an emotional oddball task in which a display of four sad faces or happy faces were shown as frequent distracter stimuli or rare deviant stimuli while high and low dysphoria participants had to detect a change of a central cross (a covert emotional oddball task). For the M170 there were no significant effects or interactions involving the group. For a later M300 component over the left occipital scalp, there were greater responses to sad than happy faces only in the dysphoric group.

More recently, Chilver et al. (2022) recorded EEG during passive viewing of emotional faces under masked (subliminal) and unmasked (conscious) conditions in a large non-clinical sample. Using multivariate linear mixed models, they reported an association between scores on the Depression Anxiety Stress Scale (DASS-42, Lovibond and Lovibond, 1995) and the N170 emotion modulation in the masked task. Higher depression/anxiety symptoms were associated with a lack of differentiation between fearful and happy faces.

Depression vulnerability has also been studied in healthy first-degree relatives of patients with major depressive disorder (MDD). Watters et al. (2018), recording EEG in a covert task of passive viewing of emotional faces found that non-relatives of MDD showed early and late emotion modulations between negative and happy faces that were attenuated in relatives of MDD. The early effects concerned frontocentral positivities (150–225 ms and 200–250 ms) rather than the posterior N170 and EPN, due to the choice of linked mastoid reference (Eimer and Holmes, 2002; Williams, 2006; Eimer et al., 2008; Buodo et al., 2015; discussed by Maffei et al., 2021). The late effect was a reduction of the parietal LPP modulation between negative and happy faces (Watters et al., 2018).

More recently, Seidman et al. (2020) recorded EEG during passive viewing of sad, happy, or neutral expressions facing forward or averted away in two groups of adolescent girls with low or high depression risk (maternal depression history). In an early (N170) and intermediate (EPN) time window, greater voltage N170, and EPN amplitudes were present in response to forward vs. averted faces only in low-risk girls. High-risk girls exhibited significantly less positive LPP responses to averted faces compared to low-risk girls. Therefore both studies concur in indicating reduced early and late emotion modulations in first-degree relatives of MDD.

### Event-Related Potential Emotion Face Processing and Attentional Task Demands

Few studies on healthy participants have investigated the influence of task demands on emotional face processing by comparing at least two tasks within-subjects (Wronka and Walentowska, 2011; Rellecke et al., 2012; Itier and Neath-Tavares, 2017; Maffei et al., 2021). Three such studies contrasted a gender discrimination task with emotional faces (covert emotion processing) with an emotion discrimination task (overt emotion processing). Wronka and Walentowska (2011) found that the right N170 was modulated by expression only in the overt task. For the intermediate stage EPN, they found a significant effect of expression but no evidence of modulation by task demands. Late components were not analyzed. Rellecke et al. (2012) reported for the N170 and the EPN main effects of emotion in the absence of task effects, while at the later stage of LPP, emotion modulations were enhanced in the overt task. Itier and Neath-Tavares (2017) also found that the N170 and EPN were modulated by emotion. N170 amplitudes were enhanced for emotional relative to neutral expressions, independent of task demand. In contrast, the EPN was affected by task demands, with greater voltages for the overt than the covert task, independent of emotion. LPP effects were not analyzed. Finally, Maffei et al. (2021) contrasted a perceptual distraction condition with task-irrelevant faces (covert emotion task) to an emotion task-relevant categorization condition (e.g., overt emotion task). As in Rellecke et al. (2012) and Itier and Neath-Tavares (2017), the N170 was enhanced by emotion irrespective of task demands, and as in Itier and Neath-Tavares (2017), the EPN amplitude was modulated by task demands, with greater voltages in the overt condition, and by emotion, with no interaction between emotion and task. As in Rellecke et al. (2012), ERP activity was modulated by emotion as a function of the task only at the late processing stage of the LPP. Combining the evidence from the available studies, Maffei et al. (2021) concluded that at the early stages of face processing (N170) affective content does not necessarily require attention. The role of voluntary attention appears to start at an intermediate stage (EPN), and fully modulate the response to emotional content in the final stage of processing (LPP), supporting recent evidence that the core and the extended part of the face-processing system act in parallel, rather than serially, and continuously exchange information (Maffei and Sessa, 2021).

### Aims of the Present Study

To our knowledge, no ERP studies to date have examined the influence of depression or dysphoria on different stages of emotional information processing as a function of covert vs. overt attentional task demands in a within subject design. Such within-subject manipulation of emotional task demands would appear very informative to test the differential effects of dysphoria on emotional face processing. The dual-process model of depression vulnerability (Beevers, 2005) would predict early effects of dysphoria on automatic stages of emotion processing likely affecting the N170 independent of top-down attentional control, and signaling early automatic attentional capture by symptom-congruent stimuli.

The main aim of the present study was therefore to address the gap in the literature by investigating the spatiotemporal dynamics of the effects of dysphoria on the covert vs. overt processing of sad, fearful, happy, and neutral facial expressions in healthy participants with high and low dysphoria. Our main predictions were that, in agreement with the dual-process model, dysphoria would differentially affect an early, automatic stage of emotion processing (the temporo-occipital N170) independent of top-down allocation of attentional demands. Secondly, the dysphoria level would not influence the intermediate (EPN) and later (LPP) stages of emotion processing independent of attentional demands or emotion category. In particular, the LPP, which reflects the allocation of processing resources and top-down attentional control to emotional expressions, would not be differentially affected by dysphoria.

Furthermore, we employed a high-density EEG electrode array and a state-of-the-art non-parametric permutation-based mass-univariate design (Maris and Oostenveld, 2007; Fields and Kuperberg, 2020) applied to all-time points and a large number of scalp electrodes, in order to fully characterize the spatiotemporal aspects of the ERP response to the emotional expressions while avoiding the constraints of an arbitrary selection of specific electrode sites or regions of interest (Luck and Gaspelin, 2017), ultimately returning significant clusters of electrodes where scalp voltage significantly differed as a function of dysphoria group, emotion, covert-overt task demands, and their interaction.

## Materials and Methods

### Participant Screening

A total of two-hundred and seventy-five undergraduate students enrolled in first- and second-year psychology courses (183 women, age = 19.13 ± 4.64) completed a screening session. After giving informed consent, participants completed a demographics questionnaire and the BDI-II (Beck et al., 1996), which is a valid and reliable measure of depression symptom severity in community, and patient samples (Beck et al., 1996), as well as college samples (3-week test re-test reliability = 0.78; Oliver and Burkham, 1979). The BDI-II uses 21 self-report items to assess the severity of recent (i.e., past 2 weeks) cognitive-affective and somatic symptoms of depression on a 0–3 scale. The total score ranges from 0 to 63. A score of 9 was employed as a cut-off score to discriminate between individuals with and without depressive symptomatology (Kendall et al., 1987). As a result, a high dysphoric group (BDI-II scored > 9) and a comparison low dysphoria group (matched on gender, education, and handedness) with minimal depression scores were recruited for the study. To ensure separation between groups, for the non-dysphoric control group we selected individuals with BDI II scores ≤ 4, and those with mid-range scores (5–8) were not included.

Participants also completed the Spielberger State-Trait Inventory (STAI; Spielberger et al., 1983). This questionnaire has 20 items rated on a 4-point scale that assess state anxiety and 20 that assess trait- anxiety. The 2-month test-retest reliability = 0.65–0.75 (Spielberger et al., 1983).

Criteria for inclusion in the study were normal or corrected-to-normal visual acuity, normal color vision, no admitted history of neurological or psychiatric disorders, drug or alcohol abuse, or learning disabilities (from the demographics screening questionnaire). Before screening and before ERP recording, participants were fully informed about all aspects of the study and signed consent forms, in compliance with the guidelines of the Simon Fraser University Research Ethics Board.

### EEG Participants

The high dysphoria group included 15 participants with mild to moderate depression symptoms [(mean BDI score = 14.53 ± 2.6; 11 women; mean age = 18.73 ± 1.58); 1.5 ± 0.86 years of post-secondary education; all right-handed]. The low dysphoria group consisted of 19 individuals with few depression symptoms [(mean BDI = 2.29 ± 1.4; 11 women; average age = 19.14 ± 1.56); 1.92 ± 1.19 years of post-secondary; all right-handed].

### Experimental Task

**FIGURE 1:**
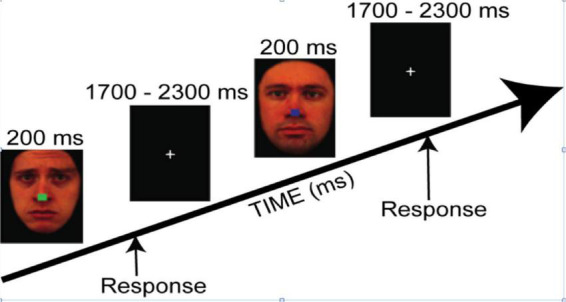
The trial sequence for both tasks, where instructions were either to categorize the color of the central square irrespective of the facial expression (i.e., the Covert task condition) or to categorize the emotional expression irrespective of the color of the central square (i.e., the Overt task condition). Reproduced with permission from Daniel Lundqvist, available at https://www.kdef.se/download-2/register.html.

The task employed colored photographs of faces from the Karolinska Directed Emotional Faces database (KDEF) (Goeleven et al., 2008, 13 males, 15 females; 4 emotions: fear, sad, happy, and neutral). Mean arousal ratings for the selected face expressions (Goeleven et al., 2008) were as follow: Fear = 3.64 ± 0.31; Sad = 3.54 ± 0.17; Happy = 3.9 ± 0.18; Neutral = 2.38 ± 0.15. All emotion categories were rated higher than Neutral pictures, *p* < 0.0001. Arousal ratings across emotions were similar except happy faces that were rated higher than sad expressions (*p* < 0.01). The faces were set on a black background and altered using Photoshop (version 10.0.1, Adobe Inc., San Jose, CA, United States) to obscure the hairline and create identical facial contours. Then each face had a small colored square (red, blue, green, or yellow) superimposed on the nose. Faces were presented for 200 ms, followed by a central fixation-cross presented for a randomly jittered inter-stimulus interval (ISI) of 1700–2300 ms (see Figure 1). The experiment was programmed using Presentation software (Neurobehavioral Systems Inc., Berkeley, CA, United States). The study included two tasks with identical stimuli but different instructions.

In the Covert emotion task, participants were asked to attend to the central square irrespective of the surrounding faces, and to choose the color as quickly and accurately as possible (blue, red, green, or yellow) by pressing one of four corresponding buttons on a gamepad controller with the index or middle finger of the left or right hand (Logitech, Romanel-sur-Monges, Switzerland).

In the Overt emotion task, participants were asked to categorize the expression of the face (happy, fearful, sad, or neutral), irrespective of the color of the central square, by pressing as quickly and accurately as possible one of the four corresponding buttons on the gamepad controller.

To help minimize eye movements, participants were instructed to keep their eyes on a central fixation (or the central square) throughout the experiment. Stimuli were displayed in a pseudo-randomized order, constrained so that no more than three stimuli with the same emotion, color, or gender were presented in a row. Participants completed a practice block and were required to reach 80% accuracy before advancing to the actual experiment. Each task (Covert vs. Overt) included 400 stimuli divided into four 5-min blocks separated by short resting periods. To control for potential confounds, button assignments relative to colors (Covert task) or emotions (Overt task) were counterbalanced between participants. The presentation order of the Overt and Covert tasks was also counterbalanced across participants. For each participant, RTs were recorded from stimulus onset and averaged for each combination of task and emotion. RTs shorter than 200 ms or longer than 2,000 ms were discarded from further analysis.

### EEG Data Acquisition

Data were collected using high-density EEG during the performance of the covert and overt face emotion tasks. Participants sat in a sound-attenuated booth with standardized ambient lighting facing a CRT monitor positioned 60 cm away from the participant’s eyes. The ActiveTwo BioSemi electrode system (BioSemi; Amsterdam, Netherlands) was used to record continuous EEG from 136 Ag/AgCl electrodes. In a survey, 130 of the electrodes were embedded in an elastic cap and positioned in a modified 10–20 equiradial layout relative to the vertex, including two sensors replacing the “ground” electrodes, i.e., the common mode sense (CMS) active electrode, and the driven right leg (DRL) (BioSemi; Amsterdam, Netherlands). Six additional external electrodes were applied: two at each lateral canthus (HEOG; for horizontal eye movements), two below each eye (VEOG; for vertical eye movements and blinks), and two over each mastoid bone. DC offset was kept below ± 25 KΩ. The continuous signal was acquired with an open passband from DC to 150 Hz and digitized at 512 Hz. The amplifier gain was fixed for each active electrode channel at 32x.

### EEG Preprocessing

The preprocessing was done in MATLAB (v2019b; RRID:SCR_001622) using functions from the EEGLab (v.2020.048; RRID:SCR_007292) and ERPLab (v8.049; RRID:SCR_009574) toolboxes. Continuous data were down-sampled to 256 Hz, high-pass filtered at 0.01 Hz, and re-referenced to the average of all channels. Following, the clean_artifacts routine in EEGLab was used with default parameters to detect bad channels and exclude them from further processing. Data were then segmented into epochs from −200 to 800 ms around stimulus onset, and lowpass filtered at 30 Hz. Excluded channels were interpolated.

Artifact detection was implemented in two steps. First, epochs contaminated by blink and saccades were detected using the pop_artstep function implemented in ERPLab, applied on periocular channels (window size = 400 ms, step size = 10 ms, amplitude threshold = 25 μV). Then, epochs with a peak-to-peak amplitude exceeding ± 100 μV in any channel were identified using the pop_artmwppth function implemented in ERPLab (window size = 200 ms, step size = 20 ms) and discarded. The average percentage of epochs retained was 94%. After preprocessing, the epochs were averaged across each condition to quantify the ERPs at each channel site and then used for statistical analyses.

### Statistical Analysis

#### Behavioral Data

Mean accuracy and Mean RT for each participant were analyzed *via* the Repeated Measures ANOVAs, with Task (2 levels: Covert and Overt) and Emotion (4 levels: Happy, Sad, Fear, Neutral) as within-subject factors, and Group (2 levels: High and Low dysphoria) as a between-subject factor. *Post hoc* paired-samples *t*-tests were run to test the main effects or interactions with a Bonferroni correction to control for family-wise error (alpha level set at *p* < 0.05).

#### Event-Related Potential Data

Statistical inference for ERP data was performed in a mass-univariate framework (Groppe et al., 2014). This approach consists of performing a statistical test for every electrode, then iteratively permuting the condition labels and performing the test again. Sufficient permutations allow the estimation of the empirical null distribution of the test statistic, which can then be used for inference. Considering the factorial design of this study, we used the factorial mass-univariate testing (FMUT) approach (Fields and Kuperberg, 2020). According to the literature on emotional face processing, we defined three *a priori* time-windows of interest: Early (140–180 ms), spanning the N170; Intermediate (200–400 ms), including the EPN; and Late (500 –750 ms), encompassing the LPP. Then, for each time window, we fit a mass-univariate ANOVA with 5000 permutations that included the predictors’ Emotion (4 levels: Fear, Happiness, Sadness, and Neutral), Task (2 levels: Covert and Overt), Group (2 levels: High and Low dysphoria) and their interactions. The multiple comparisons problem was handled using a cluster-based approach (Maris and Oostenveld, 2007). Noting that the components of interest are all characterized in the literature as having posterior topographic distributions, we opted to restrict the analysis to the mean activity of the posterior part of the scalp, improving statistical power. When the FMUT revealed a significant effect, it was further explored using *post hoc* mass-univariate t-tests, run with 5,000 permutations, and corrected for multiple comparisons using the cluster-based approach (Groppe et al., 2014).

## Results

### Demographic Data

The high and low dysphoric groups did not differ on any of the matched variables (sex, education, handedness, and age). The high group, however, scored above the cut-off (>35) on the STAI-state anxiety measure (average = 39 ± 9.77), and the low group did not [average = 28.73 ± 6.63; *t*(29) = −3.37, *p* = 0.002, *d* = 1.23]. Additionally, the high group scored above the cut-off (>40) on the STAI-trait measure (average = 45.00 ± 9.59), whereas the low group did not [average = 31.87 ± 7.64; *t*(29) = −4.173, *p* = 0.000, *d* = 1.514]. In line with large-scale studies conducted on these measures in undergraduate students (e.g., Gotlib, 1984) the BDI was strongly correlated with the STAI-trait (*r* = 0.77, *p* = 0.000, *d* = 2.41), and STAI-state (*r* = 0.52, *p* = 0.003, *d* = 1.218) measures.

### Behavioral Data

#### Accuracy

Participants of both groups were highly accurate in both the overt and covert tasks (all participants scored at least 85% or above). A repeated measures ANOVA revealed significant main effects of Task [*F*_(1,32)_ = 53.92, *p* < 0.001, η*_*p*_^2^* = 0.63] and Emotion [*F*_(3,96)_ = 22.07, *p* < 0.001, η*_*p*_^2^* = 0.41]. These main effects were qualified by a significant Task × Emotion interaction [*F*_(3,96)_ = 21.42, *p* < 0.001, η*_*p*_^2^* = 0.4]. Critically, the main effect of Group and all interactions involving Group were not significance (for all, *F* < 0.44, *p* > 0.62).

*Post hoc* paired-samples *t*-tests on the Task × Emotion interaction revealed that: Participants were less accurate when responding to all overt relative to covert stimuli (*p* < 0.001), except for happy expressions. There were no differences between emotions during the Covert task. In contrast, during the Overt task, happy face responses were more accurate than all other expressions (*p* < 0.001), and fearful and sad faces were recognized less accurately than neutral expressions (*p* < 0.001 and *p* = 0.002, respectively). See details in Table 1.

**TABLE 1:** Mean accuracy and mean reaction time (in milliseconds) for the high and low dysphoria groups as a function of Task and Emotion.

	Happy	Fearful	Sad	Neutral
**Percent correct (SD)**				
**Covert**				
Low	97 (3.0)	97 (2.9)	96 (3.1)	96 (2.4)
High	97 (3.0)	97 (2.7)	96 (3.5)	97 (2.5)
Total	97 (3.0)	97 (2.8)	96 (3.3)	97 (2.5)
**Overt**				
Low	97 (2.2)	88 (7.2)	89 (6.3)	94 (4.8)
High	98 (2.4)	86 (12.0)	88 (10.0)	94 (6.4)
Total	97 (2.4)	87 (9.5)	89 (8.2)	94 (5.4)
**Mean RT (SD)**				
** Covert**				
Low	734.46 (72.74)	738.91 (76.17)	739.79 (61.90)	733.39 (69.83)
High	698.57 (71.58)	697.36 (69.43)	696.95 (70.44)	699.40 (74.26)
Total	718.63 (73.39)	720.58 (75.15)	720.89 (68.27)	718.40 (72.75)
** Overt**				
Low	775.50 (74.32)	936.62 (92.38)	900.39 (81.38)	852.99 (83.75)
High	736.98 (60.06)	888.77 (52.04)	853.28 (70.94)	819.48 (54.72)
Total	758.50 (70.14)	915.51 (79.91)	879.61 (79.44)	838.21 (73.36)

#### Reaction Time

The repeated-measures ANOVA returned significant main effects for Task [*F*_(1,32)_ = 231, *p* < 0.001, η*_*p*_^2^* = 0.88] and Emotion [*F*_(3,96)_ = 65.44, *p* < 0.001, η*_*p*_^2^* = 0.67], both qualified by a significant Task × Emotion interaction [*F*_(3,96)_ = 63.35, *p* < 0.001, η*_*p*_^2^* = 0.66]. Critically, the main effect of Group (*p* > 0.05) and all interactions involving Group were far from significance (for both, *F* < 0.04, *p* > 0.85).

*Post hoc* paired-samples *t*-tests on the Task × Emotion interaction revealed that: Participants were slower when responding to all overtly presented relative to all covertly presented emotions (*p* < 0.001), with the exception of happy expressions. There were no differences between emotions in the Covert task; In contrast, for the Overt task, happy faces were responded to more quickly than all other expressions (*p* < 0.001), and fearful and sad faces were slower to categorize than neutral expressions (for both *p* < 0.001), while they were not dissimilar among each other. See details in Table 1.

### Event-Related Potentials

#### Early Time Window (140–180 ms)

The FMUT analysis returned significant main effects of Task (F cluster mass = 229.91, *p* = 0.02), Emotion (F cluster mass = 436.37, *p* < 0.001), and Group (F cluster mass = 83.21, *p* = 0.03). For the main effect of the Task, the Overt vs. Covert contrast showed that there was greater negative voltage in the N170 time window for the Covert than the Overt task, resulting in a positive cluster with the bilateral inferior temporo-occipital distribution.

**FIGURE 2:**
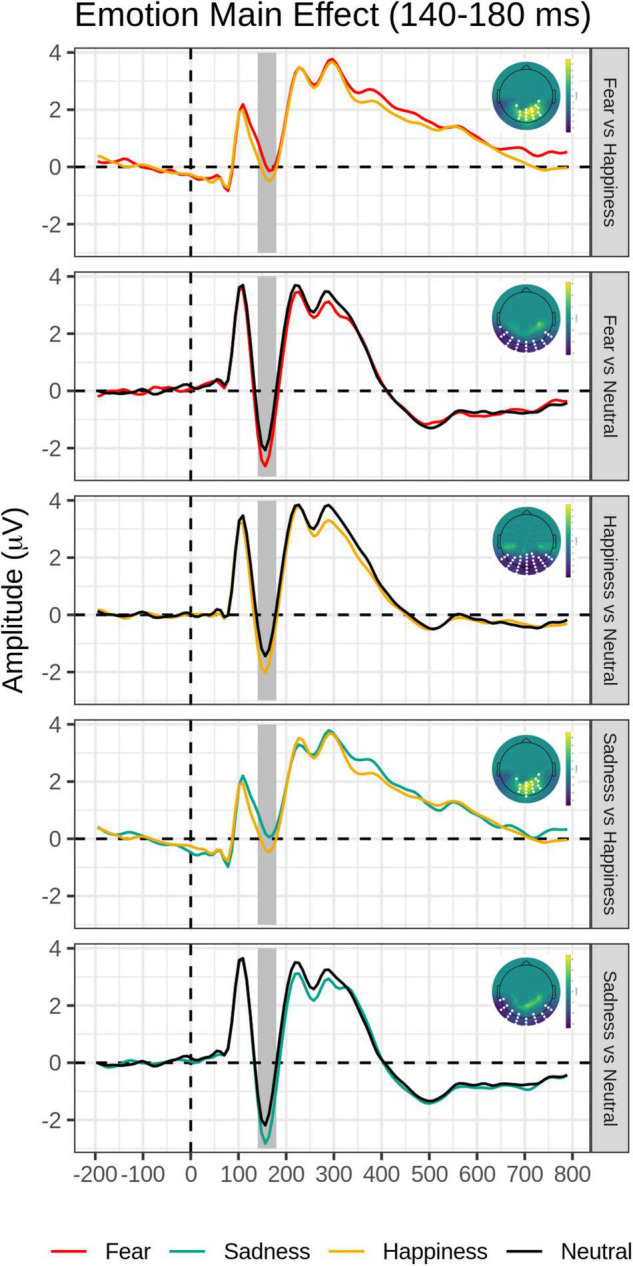
Emotion main effect in the early time window (140–180 ms): Grand-average waveforms of the electrodes included in the significant negative or positive clusters and the scalp topography of the significant clusters (with the significant electrodes highlighted in white). Covert and Overt tasks are combined.

*Post hoc* pairwise contrasts on the Emotion main effect showed that all emotion expressions (happy, afraid, and sad) yielded greater N170 voltage than neutral faces with a negative cluster over the bilateral inferior temporo-occipital scalp. Furthermore, the contrasts between Sad vs. Happy and Fearful vs. Happy faces both revealed significant positive clusters (greater N170 for Happy Faces), with a more dorsal distribution over the parietal scalp (see Table 2 and Figure 2).

**TABLE 2:** *Post hoc* pairwise mass-univariate tests showing the significant negative and positive clusters in the early time window (140–180 ms).

	Mean A (SD)	Mean B (SD)	T mass	*P*-value
**Emotion main effect**				
Fearful–Happy*	0.23 (0.41)	−0.17 (0.40)	60.04	0.002
Fearful–Neutral	−1.82 (0.59)	−1.24 (0.57)	−102.13	<0.001
Happy–Neutral	−1.34 (0.54)	−0.77 (0.53)	−146.07	<0.001
Sad–Happy*	0.37 (0.40)	−0.16 (0.41)	85.58	<0.001
Sad–Neutral	−1.99 (0.58)	−1.36 (0.56)	−105.27	<0.001
**Task main effect**				
Overt–Covert*	−1.43 (0.59)	−2.15 (0.63)	64.53	0.004
**Emotion × Group interaction (high vs. low dysphoria)**				
Fearful	−3.62 (0.91)	−1.12 (0.91)	−31.89	0.03
Happy	−3.32 (0.80)	−0.89 (0.80)	−36.51	0.02
Neutral	−2.98 (0.85)	−0.63 (0.85)	−32.66	0.03
Sad	−3.89 (0.99)	−1.19 (0.99)	−27.49	0.054
**Emotion × Group Interaction (high dysphoria group only)**				
Fearful–Happy*	0.25 (0.53)	−0.3 (0.54)	46.42	0.005
Fearful–Neutral	−2.44 (0.86)	−1.75 (0.85)	−88.3	0.004
Sad–Happy*	0.55 (0.49)	−0.05 (0.44)	41.84	0.01
Sad–Neutral	−2.5 (0.88)	−1.75 (0.82)	−114.22	<0.001
Happy–Neutral	−1.98 (0.74)	−1.19 (0.76)	−127.57	<0.001
**Emotion × Group interaction (low dysphoria group only)**				
Fearful–Neutral	−1.06 (0.75)	−0.44 (0.72)	−51.3	0.008
Sad–Happy*	0.67 (0.58)	0.09 (0.62)	70.27	<0.001
Sad–Neutral	−1.34 (0.65)	−0.67 (0.66)	−53.26	0.006
Happy–Neutral	−1.02 (0.76)	−0.53 (0.73)	−78.05	0.003

*Positive cluster, all other clusters were negative.

The main effect of the Group was explained by significantly greater N170 amplitude in the High than the Low dysphoria group over the left inferior temporo-occipital scalp. The main effects of Emotion and Group were qualified by the significant Emotion by Group interaction (F cluster mass = 42.09, *p* = 0.03).

**FIGURE 3:**
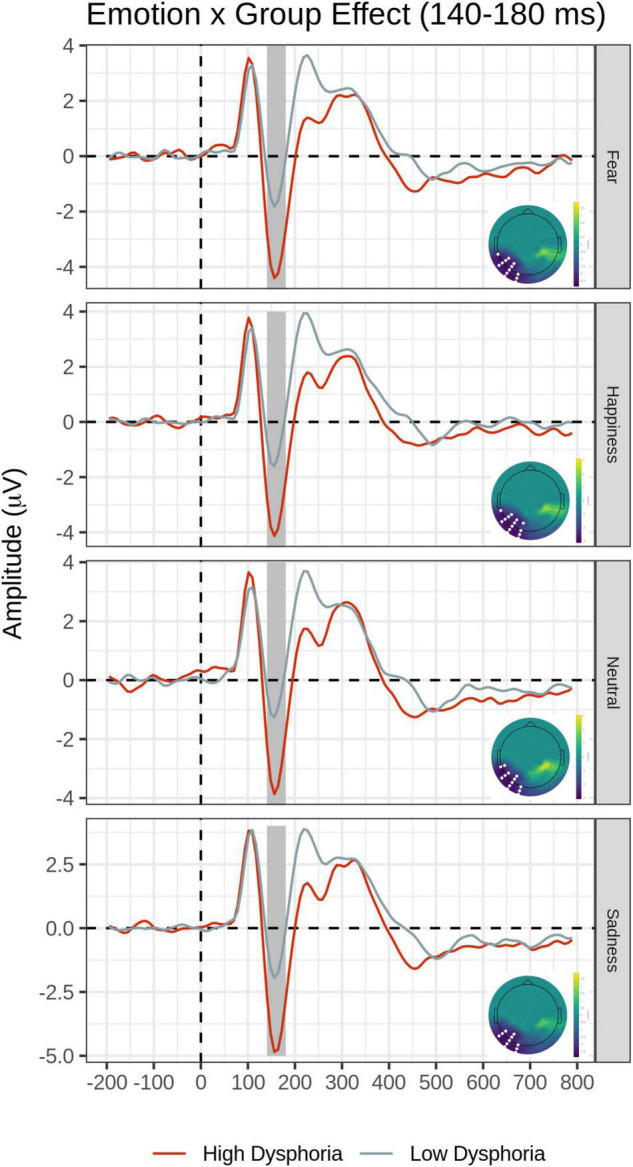
Group × Emotion interaction for the early time window (140–180 ms). In each row, average waveforms of the High and Low dysphoria groups for each emotion and the scalp topography of the significant clusters for the High vs. Low dysphoria group contrast for each emotion (with the significant electrodes highlighted in white). Covert and Overt tasks are combined.

*Post hoc* pairwise contrasts were carried out to disentangle such interaction (see Table 2). First, within-group pairwise contrasts revealed similarly significant N170 emotion-enhancement effects across experimental groups, but with one difference. Comparisons of each emotion expression relative to Neutral (Happy vs. Neutral, Fear vs. Neutral, Sad vs. Neutral) yielded similar significant negative clusters of greater N170 amplitude over the bilateral inferior temporo-occipital scalp. Furthermore, the contrast between Happy and Sad faces revealed a similar significant negative cluster (greater N170 for Happy Faces), with a more dorsal distribution over the parieto-occipital scalp (see Figure 2). Unique to the High dysphoria group was a significant contrast between Happy and Fearful faces (less N170 to fearful faces) with a negative cluster with a dorsal distribution over the parietal scalp. Between groups, pairwise contrasts showed that there was significantly greater voltage amplitude in the N170 time window for the High dysphoria group relative to the Low dysphoria group for all expressions (with the sad faces approaching significance) with negative clusters with a scalp distribution over the left lateral inferior temporoparietal scalp (see Table 2 and Figure 3). Please note that no significant clusters were detected for the Task × Emotion interaction in the early time window.

#### Intermediate Time Window (200–400 ms)

The FMUT analysis revealed significant main effects of Task (F cluster mass = 1609.4, *p* < 0.001) and Emotion (F cluster mass = 55.19, *p* = 0.003).

**FIGURE 4:**
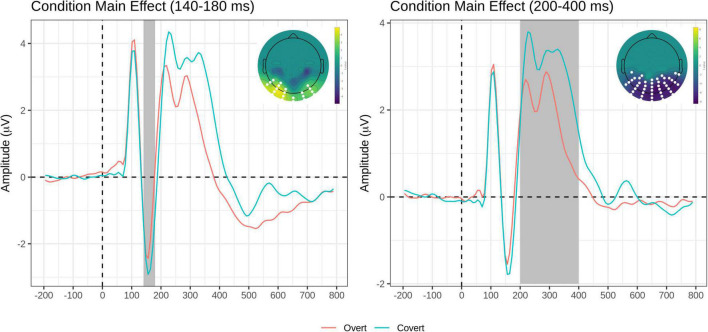
Task main effects for the early time window (140–180 ms, left) and the intermediate time window (200–400 ms, right). On each panel, grand-average waveforms for the Overt (red) and Covert (teal) task for the electrodes included in the significant clusters (highlighted in white on topographical maps); scalp topography of the Overt vs. Covert contrast for the significant clusters (again, the significant electrodes are highlighted in white). The Emotion factor is collapsed.

For the main effect of Task, the contrast Overt vs. Covert revealed a large negative cluster like the EPN, with greater negative voltage for Overt than Covert expressions, but extended to a broader region over the posterior scalp, including bilateral occipital and parietal sites (see Table 3, top and Figure 4, right).

**TABLE 3:** *Post hoc* pairwise mass-univariate tests showing the significant negative and positive clusters in the intermediate time window (200–400 ms) and late time window (500–750 ms).

	Mean A (SD)	Mean B (SD)	T mass	*P*-value
**Intermediate time window (200–400 ms)**				
**Emotion main effect**				
Sad–Happy*	3.83 (0.47)	3.55 (0.48)	28.81	0.03
Happy–Neutral	2.93 (0.47)	3.29 (0.48)	−71.39	0.002
**Task main effect**				
Overt–Covert	1.87 (0.42)	3.00 (0.44)	−256.96	<0.001
**Late time window (500–750 ms)**				
**Emotion × Task (overt task only)**				
Fearful–Happy	−0.66 (0.24)	−0.21 (0.20)	−34.48	0.02
Happy–Neutral	1.14 (0.27)	1.64 (0.26)	−26.95	0.04
Sad–Happy	−0.94 (0.27)	−0.48 (0.23)	−47.38	0.004
Sad–Neutral	1.0 (0.27)	1.36 (0.25)	−35.5	0.02

*Sad vs. Happy was the only positive cluster, all other clusters were negative.

**FIGURE 5:**
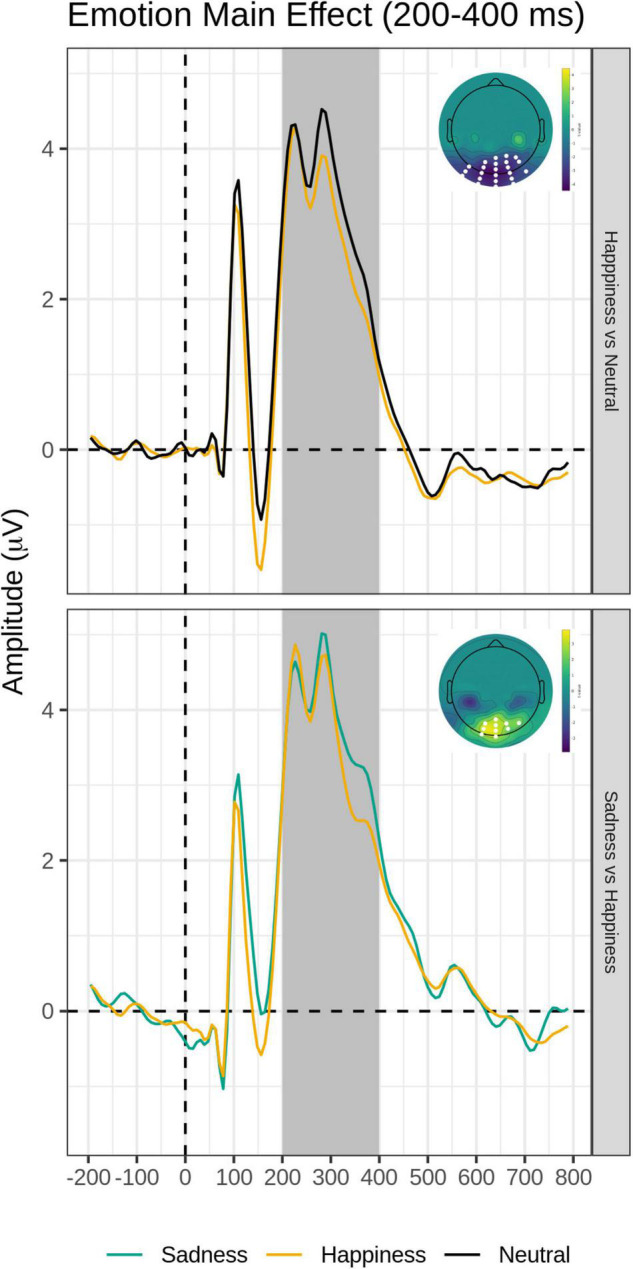
Emotion main effect of the intermediate time window (200–400 ms). Grand-average waveforms for Happy vs. Neutral (top) and Sad vs. Happy (bottom) contrasts for each electrode included identified by the significant negative or positive clusters. Scalp topography maps of the relevant contrasts displaying the significant clusters (with the significant electrodes highlighted in white). Covert and Overt tasks are combined.

The *post hoc* pairwise contrasts on the Emotion main effect showed that happy expressions were associated with increased negativity (greater EPN) relative to neutral faces over the bilateral inferior parieto-occipital scalp. In addition, the contrast between Sad and Happy faces revealed a significant positive cluster (greater EPN for Happy Faces), with a more restricted distribution over more dorsal and central parietal scalp (Table 3, top and Figure 5).

There were no significant clusters detected for the Task × Emotion interaction. Importantly, there were no significant clusters detected for the Group’s main effect nor any interactions involving the group.

#### Late Time Window (500–750 ms)

The FMUT analysis revealed significant main effects of Emotion (F cluster mass = 43.83, *p* = 0.03) and Task (F cluster mass = 728, *p* < 0.001), and a significant Emotion by Task interaction (F cluster mass = 39.72, *p* = 0.04). This pattern of results was further explored by *post hoc* mass-univariate *t*-tests on the Task × Emotion interaction (Table 3, bottom). Unsurprisingly, larger amplitudes were evident for faces in the Overt relative to the Covert task (not shown in Table 3, bottom).

**FIGURE 6:**
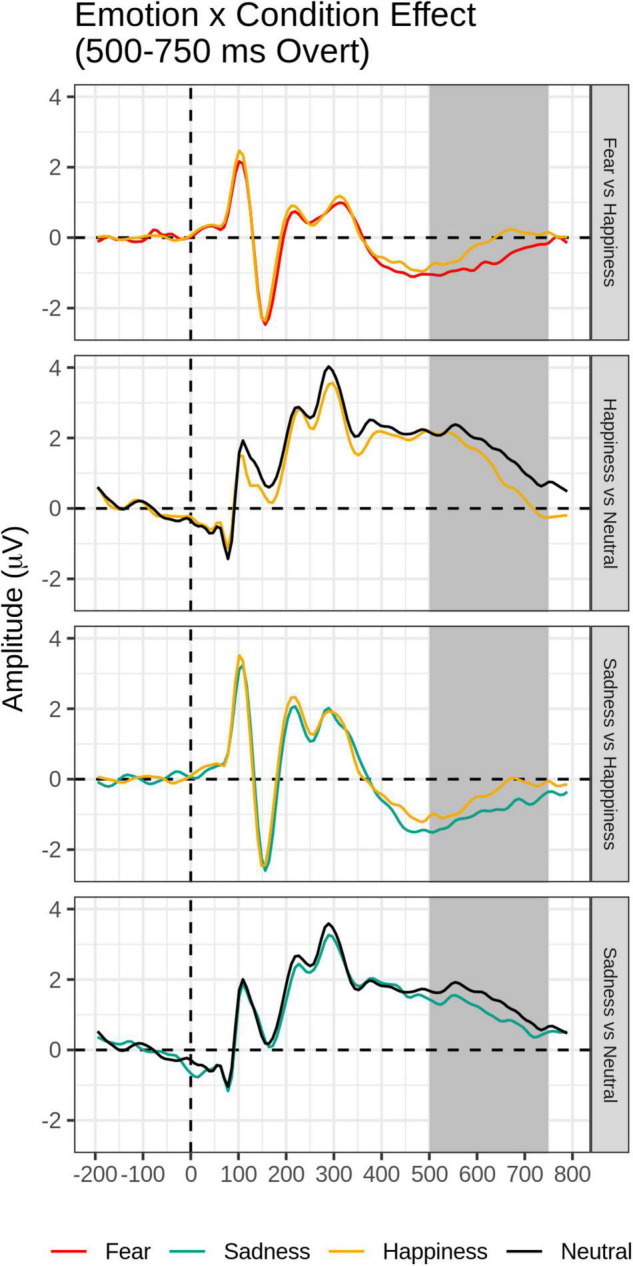
Emotion × Task effect for the late time window (500–750 ms): In each panel, grand-average waveforms of the electrodes are included in the significant negative or positive clusters during the overt condition only.

For the Overt task, Fear and Sad expressions elicited significantly greater LPP voltages compared to Happy expressions. Sad faces and Happy faces gave rise to larger LPP amplitudes relative to Neutral expressions (Table 3, bottom and Figure 6). Significant clusters were all negative, chiefly due to the choice of restricting the F-MUT analysis to the mean activity of the posterior part of the scalp, which left out dorsal parietal and central regions where the LPP typically displays positive polarity. Critically, as for the preceding intermediate window, there was no main effect or interactions involving Group.

## Discussion

Behavioral and ERP correlates of the influence of dysphoria on covert vs. overt processing of happy, fearful and sad vs. neutral facial expressions were investigated applying a non-parametric mass univariate approach. No behavioral differences were found as a function of dysphoria. As predicted by the main hypothesis, electrophysiologically, dysphoria impacted the early automatic stage of emotional processing, with two effects: a significant N170 voltage amplitude increase over the left inferior temporo-occipital scalp in the high vs. low dysphoria group, and a significant N170 voltage difference between happy and fearful expressions only present in the high dysphoria group. Both effects were irrespective of attentional task demands.

As also hypothesized, no effects of dysphoria were present at intermediate or later processing stages: neither the emotion-sensitive EPN nor the emotion-sensitive LPP.

The left-lateralized N170 enhancement in the high dysphoria group suggests a different modulatory mechanism from those responsible for top-down attentional control or emotional regulation.

### Behavioral Results

Regardless of the experimental group, all participants were slower and less accurate during the overt vs. covert task. Additionally, there were no behavioral differences in RT between emotion categories in the covert emotion task, as previously found in other studies where emotion was task-irrelevant (e.g., gender discrimination studies: Wronka and Walentowska, 2011; Rellecke et al., 2012; Itier and Neath-Tavares, 2017).

In the overt task, happy faces were explicitly categorized more quickly and accurately relative to all other expressions, a finding reported before in the literature (Calvo and Beltrán, 2014) and consistent with a body of behavioral research showing a recognition advantage for happy faces compared to the other basic expressions (Beltrán and Calvo, 2015).

Of relevance here was the finding that higher dysphoria levels did not produce measurable behavioral RT effects for covertly or overtly presented sad or happy faces. A similar lack of behavioral effects was reported in a previous ERP study of the effects of dysphoria on covert emotion face processing (although note that this study did not include an overt task condition; Buodo et al., 2015).

Without electrophysiological results, the behavioral data would support a lack of mood-congruent attentional bias in depression and suggest that depression does not influence early, automatic attentional capture of depression-related stimuli (e.g., Gotlib and Cane, 1987; Bradley et al., 1997; Mogg and Bradley, 2005; Gotlib and Joormann, 2010), and may not influence later stages of processing.

### Event-Related Potential Results

#### Early Time Window (140–180 ms)

An N170 voltage amplitude enhancement over the bilateral inferior temporo-occipital scalp was found for each emotional expression relative to neutral expressions, as typically reported in the literature (e.g., Batty and Taylor, 2003; Schupp et al., 2004, 2007; Itier and Neath-Tavares, 2017) and for happy expressions relative to sad expressions over the more dorsal and central parieto-occipital scalp (Maffei et al., 2021; again see Figure 2 and Table 2).

A novel result of the present study is the finding that N170 amplitude over the bilateral inferior temporo-occipital scalp was *greater* for the covert (i.e., perceptual distraction) task than in the overt (i.e., categorization) task, independent of emotion, and independent of the group.

Of the previous ERP studies contrasting gender discrimination (covert) to an emotional discrimination (overt) task, Wronka and Walentowska (2011) reported a significant task by emotion interaction, with the right N170 being enhanced by emotion in the overt task, concluding that it probably reflected voluntary attentional control. Rellecke et al. (2012) and Itier and Neath-Tavares (2017) both reported no significant task or emotion × task interaction effects. Interestingly, the perceptual discrimination task used as a covert task in the present study involved greater attentional demands and response choice requirements (four options) than the gender discrimination task (two options) employed in the three previous studies, yet the N170 voltage amplitude in response to task-irrelevant faces was greater than in the overt task, despite the increased task demands. Perhaps the N170 covert advantage requires a larger sample size to manifest, like the one (*n* = 35) in the present study. In fact, a previous report from our group using the same covert and overt tasks and the same F-MUT approach in an independent, smaller size group (*n* = 15) did not reveal the effect (Maffei et al., 2021).

While there is no supporting evidence from previous ERP literature, fMRI studies have found that partially distinct networks support the processing of covert and overt presentations of emotion. Specifically, when emotional stimuli, particularly fearful faces, were presented covertly, subcortical limbic regions, including the amygdala, thalami, hippocampi, and paralimbic cortical regions [ventrolateral and dorsomedial prefrontal cortex (PFC), including the anterior cingulate cortex (ACC)], were recruited (Vuilleumier et al., 2001; Williams et al., 2005; Scheuerecker et al., 2007). In contrast, when stimuli were presented overtly, activation from these subcortical regions was absent or recruited to a significantly lesser extent (Critchley et al., 2000; Scheuerecker et al., 2007) and neocortical regions (dorsolateral PFC and dorsal ACC) were recruited to a greater extent (Scheuerecker et al., 2007). Could enhanced N170s in the covert task reflect greater/more efficient bottom-up signaling from the amygdala or other subcortical emotional regions?

Caution should be exerted in drawing inferences about underlying brain structures since no source analysis was carried out in this study. A follow-up functional connectivity study in source space may provide clues concerning the dynamic reconfiguration of functional connections among key nodes in the face-processing systems between covert and overt tasks.

More relevant here was another novel finding concerning the main aim of the present study. Our main prediction that dysphoria would affect face processing at the early stages of face perception and would not be affected by top-down attentional control was upheld. Although we did not find a typical pattern of mood-congruent attentional bias, the high dysphoria group uniquely displayed two effects: A significant emotion modulation in the contrast between happy and fearful faces, not present in the low dysphoria group, and a significant left-sided increase of N170 amplitude compared to the low dysphoria group, present for all expressions, included the neutral faces, albeit it only approached significance for sad faces. Both effects took place irrespective of covert vs. overt emotional processing and top-down attentional control.

In previous ERP studies employing emotional faces in clinically depressed patients, Dai and Feng (2012), as well as Chen et al. (2014), reported smaller N170 amplitudes independent of expression relative to healthy controls, with the smallest amplitudes in recurrent-depression patients (Chen et al., 2014). However, other studies have found no significant N170 voltage differences (Foti et al., 2010; Jaworska et al., 2012). Negative bias has also been reported as underlying higher N170 voltage amplitude for sad faces relative to happy and neutral faces in depressed participants (Chen et al., 2014; Zhao et al., 2015; Dai et al., 2016).

As reviewed in our introduction, a number of studies exploring the effects of dysphoria in healthy populations on ERPs to emotional faces, have reported N170 effects. Buodo et al. (2015) employing a covert face task similar to the one of the present study, and recording with a linked mastoid reference, found no N170 effects but reported an increase of an early frontocentral P200 to sad relative to neutral facial expressions independent of dysphoria level. Dai et al. (2016), used an overt valence rating task and reported no N170 amplitude differences as a function of emotion or group. The MEG face study conducted by Xu et al. (2018) using a passive oddball task in dysphoria found no significant effects for the M170 nor interactions involving the group.

Chilver et al. (2022) study found an association between scores on the Depression Anxiety Stress Scale (DAS-42) and the N170 emotion modulation only in a masked (subliminal) passive viewing task. Higher symptoms were associated with a lack of differentiation between fearful and happy faces. Given the lack of effects in the unmasked (conscious) condition, and previous research from the same research group associating trait-anxiety to N170 enhancements to fearful faces under masked conditions and trait-depression to N170 enhancements in the conscious task (Williams et al., 2007; see also Watters et al., 2018), we speculate that the effect in Chilver et al.’s (2022) study was driven by anxious symptoms causing hypervigilance to threat. It is also worthwhile to note that in our study the happy vs. fear contrast was the one explaining the group by emotional interaction, being significant in the high dysphoria group.

Watters et al. (2018), employing the same covert passive viewing task in first-degree relatives of MDD patients under masked and unmasked conditions using a linked mastoid reference, found no effect on the N170 but the early frontocentral positivity (150–225 ms) showed an emotion modulation (negative vs. happy) only in the non-risk group, being attenuated in the high-risk group. A similar finding was reported by Seidman et al. (2020) in a passive viewing task with expressions directed forward or averted away in adolescent girls with maternal depression history. N170 amplitudes were greater for forward vs. averted faces only in the group without maternal depression history, while such difference was absent in the maternal depression group.

To summarize, all of the face emotion studies in subclinical depression [with the exception of Dai et al. (2016)] have employed a covert emotion task. Results have been mixed: some have reported no effects of dysphoria on the N170 (Buodo et al., 2015; Dai et al., 2016; Xu et al., 2018), and others have found an attenuation of emotion modulations in the subclinical depression group relative to controls (Watters et al., 2018; Seidman et al., 2020; Chilver et al., 2022). None of such studies have manipulated top-down attentional demands, contrasting covert to overt emotional tasks.

Therefore, our study is the first to report that high dysphoria individuals relative to low dysphoria individuals display greater amplitude left-lateralized N170s to emotional faces over the temporo-occipital scalp, independent of covert-vs.-overt attentional demands and present for all emotion categories, including neutral expressions, although for sad expressions only approached significance.

Previous ERP face studies in healthy individuals have indeed often reported significantly larger N170 amplitudes over right than left lateral posterior sites (Bentin et al., 1996; Rossion and Jacques, 2012; Wronka and Walentowska, 2011; Itier and Neath-Tavares, 2017). This effect appears to reflect a right hemisphere specialization for early structural encoding of face features (Bentin et al., 1996) but also greater N170 emotion modulation (Calvo and Beltrán, 2014; Itier and Neath-Tavares, 2017). Why is the N170 to faces left-lateralized among dysphoric individuals?

A first interpretation should take into account reports that both in adults and in infants N170 modulations over left-sided electrodes may be associated with a more feature-based rather than holistic/configural face processing strategy (Scott and Nelson, 2006). Calvo and Beltrán (2014) presented emotional faces in three formats (whole face, upper half visible, and lower half visible) in an overt emotion categorization task. They found that the right-hemisphere dominant N170 (150–180 ms) was modulated by expression of whole faces, but not by separate halves, suggesting that expression encoding (N170) may require holistic processing in the right hemisphere. In contrast, the mouth region of happy faces enhanced the left temporo-occipital N170 activity and suggested that analytical or part-based processing of the salient smile is early and left-lateralized, possibly accounting for the behavioral happy face advantage (Calvo and Beltrán, 2014).

We posit that the left-sided N170 effect in our study is a feature-based face processing strategy among dysphoric individuals. Incidentally, in the high dysphoria group, happy faces elicited significantly larger N170 amplitudes than any other category of emotion, and unique to this group was a significant increase relative to fear expressions. In the literature, the fear-related N170 enhancement is the most robust N170 emotion effect and is typically right-lateralized and relies on holistic processing. Corroborating evidence for this result and its interpretation comes from an ERP study using an emotional goNoGo task with sad, happy, and neutral faces in children and adolescents with and without clinical depression. Employing a similar non-parametric topographic approach this study found that the topography of the N170 showed the typical right-lateralization in control children. In contrast, *t* maps indicated more negativity over the left hemisphere in children with major depression for all expressions, who appeared to lack the right-hemispheric specialization. Similar to us, they proposed that children with major depression may employ a different strategy in their recognition of faces that are less focused on the face as a whole but on particular features like eyes or mouth (Grunewald et al., 2015).

Another support for our interpretation comes from studies exploring the effects of emotional states and affect-related personality traits on visual perceptual processing. Employing hierarchical Navon stimuli to reveal global or local biases in visual perception, positive emotional states have been found to facilitate a broad attentional focus thereby enhancing global processing (Gasper and Clore, 2002; Fredrickson and Branigan, 2005; Rowe et al., 2007; Johnson et al., 2010). In contrast, negative emotions, such as those associated with anxiety or depression, have been related to a narrower attentional focus, therefore, facilitating local visual features (Basso et al., 1996). Curby et al. (2012) induced positive, negative, or neutral emotions through video clips and measured holistic face processing through a composite face task before and after the mood induction. Emotional state significantly modulated face processing style, with the negative emotion induction leading to decreased holistic processing.

Taken together, we speculate that both dysphoria effects, e.g., greater N170 voltage for happy relative to fearful expressions in the high dysphoria group, and greater voltage amplitudes over left posterior sites relative to low dysphoria individuals for all emotion categories, may be explained within the same theoretical account, i.e., the impact of depressive symptomatology on face processing producing a shift toward processing more feature-based aspects of faces rather than global/holistic face processing supported by the right occipitotemporal area. The present study did not manipulate feature-based vs. holistic processing, but happy expressions (recognized chiefly through the mouth region) compared to other expressions (which require integration of information from the eyes and the mouth regions, like fear and sadness)-beyond an overall behavioral and early ERP advantage relative to other expressions, gave rise to significantly greater N170s than expressions of fear exclusively in the high dysphoria group.

Future ERP studies manipulating featural vs. holistic face processing in dysphoric and depressed individuals appear to be necessary to confirm this intriguing hypothesis, for example using a composite face task.

An alternative interpretation may consider ERP studies showing that while the N170 elicited by emotional faces is typically right-lateralized (Bentin et al., 1996; Calvo and Beltrán, 2014), when negative valence faces were shown in a congruent surrounding *emotional context*, e.g., a fearful face within a fear-inducing scene (like a car accident) the N170 was left-lateralized. Such a result was not present for happy or neutral faces in happy or neutral contexts. The authors concluded that their participants were using the congruous environmental context (i.e., the scene), to rapidly discriminate/categorize negatively valenced faces at early information-processing stages (Righart and de Gelder, 2006, 2008). Although the environmental context is an exogenous factor, and dysphoric symptoms are an endogenous factor, it is possible, at least in theory, that a similar interpretation could be used here. Perhaps the high dysphoria group made use of an *internal* context (their negatively valenced depression-related schemas), to bias the early-stage processing of all stimuli in the task. In terms of possible mechanisms, the left-lateralization could be interpreted as modulation of ongoing visual processing by endogenous, perhaps amygdala or insula-initiated, gain mechanisms in the left prefrontal cortex (Vuilleumier, 2005; Pourtois et al., 2013). Thus, the lateralized N170 may reflect feedback connections between frontal “affective” regions and visual cortices, allowing for the enhancement of sensory processing.

In fMRI studies of cognitive reappraisal of emotion, down-regulation of negative affect engages cognitive control regions, in particular, left dorsolateral PFC (DLPFC) and ventrolateral PFC [see a meta-analysis in Buhle et al. (2014)]. Left DLPFC is also the preferred target of neuromodulation (rTMS) for the treatment of drug-resistant depression, where activity in left DLPFC is upregulated (Somani and Kar, 2019).

EEG functional connectivity approaches in source space may be useful to shed light on how the left occipitotemporal area may be modulated by top-down frontal control regions independently from the top-down attentional mechanisms in dysphoric individuals.

#### Intermediate Time Window (200–400 ms)

Unlike the early time window, for the intermediate epoch containing the EPN, there was a main effect of task demands, with greater voltage in the Overt than Covert emotion task, as well as a main effect of Emotion, but no evidence that the EPN emotion enhancement was affected by covert-vs.-overt processing. Critically for the present study, there were no main effects nor interactions involving the dysphoria group.

An EPN voltage amplitude modulation over the bilateral temporo-occipital scalp was found for happy expressions relative to neutral expressions, as previously reported in the literature (e.g., Schupp et al., 2003, 2004, 2007; Rellecke et al., 2012) and for happy expressions relative to sad expressions over the more dorsal and central parieto-occipital scalp (see Figure 3 and Table 3). This is consistent with the conclusion that the EPN may reflect enhanced processing of emotional salience of stimuli in general, rather than a perceptual encoding mechanism (Schupp et al., 2006).

An EPN voltage amplitude enhancement for the Overt vs. Covert task extended to a broader region over the posterior scalp, including bilateral occipital and parietal sites (see Table 3 and Figure 4). This effect has been previously found in two studies manipulating task demands within-subjects, suggesting that in the EPN time window, cognitively mediated top-down attentional control modulates neural activity, presumably reflecting the depth of processing of all face stimuli in the explicit emotion discrimination task, independent of emotion category (Itier and Neath-Tavares, 2017; Maffei et al., 2021).

More critically for this study, and in agreement with our hypothesis that dysphoria would differentially affect only early automatic processing of emotional faces, there were no main effects of dysphoria group nor any interactions involving group as a function of task demands or emotion category.

Of the previous EEG emotion studies of dysphoria, results on the EPN time window have been mixed. Several have reported no effects (Buodo et al., 2015; Xu et al., 2018; Chilver et al., 2022). In the two studies on high-risk depression relatives, Watters et al. (2018) reported attenuated frontocentral N200 to negative vs. happy faces (co-occurring with the EPN) for first-degree relatives compared to non-relatives, and Seidman et al. (2020) attenuated EPN to forward vs. averted faces in daughters with maternal depression compared to girls without depression history.

Worth commenting upon is Dai et al. (2016) study, the only to use an overt task of valence rating. This study reported a significant difference between high-dysphoria and control groups. The authors reported that a posterior P2 (150–320 ms) was more positive for happy faces in the dysphoric relative to the control group. In fact, its timing and scalp distribution were that of the EPN, an emotion difference wave that overrides the posterior P2, which would indicate, as in the previous two studies, reduced EPN for happy faces in dysphoria.

As mentioned earlier, none of such studies have manipulated top-down attentional demands, contrasting covert to overt emotion tasks.

#### Late Time Window (500–750 ms)

The main effect of task demands continued in the late time epoch, with greater amplitude LPP to overtly processed than covertly processed facial expressions. This is not surprising given the fact that the LPP is thought to reflect the increased allocation of processing resources to relevant stimuli (Schupp et al., 2006; Hajcak et al., 2010) and that the LPP emotion effects are attenuated by top-down regulation strategies, such as suppression and reappraisal (Hajcak et al., 2010).

Importantly, at this late stage, the effect of task demands varied as a function of emotion. While no LPP voltage differences were present among the emotions in the covert emotion task, for the Overt task, paralleling the RT findings, Fear and Sad expressions elicited significantly greater LPP voltages compared to Happy expressions, and Sad faces and Happy faces gave rise to larger LPP amplitudes relative to Neutral expressions (see also Maffei et al., 2021). The results of the study suggest that the allocation of processing resources and top-down attentional control to emotional expressions in the overt emotion task operates at the late stage of the LPP. Two of the previous ERP face studies manipulating task demands reported on late effects and reported similar findings (Rellecke et al., 2012; Maffei et al., 2021).

More critically for this study and in agreement with our hypothesis that dysphoria would differentially affect only early automatic processing of emotional faces, there were no main effects of dysphoria group nor any interactions involving group as a function of task demands or emotion category on the LPP time window. Noteworthy is the finding that Sad and Fearful expressions produced larger LPP effects relative to Happy faces, and that Sad Faces yielded greater LPP amplitudes than neutral faces only when top-down attentional control was exerted, i.e., when people paid attention to the personal salience of facial expressions. However, consistent with our predictions, these effects did not differ between dysphoria groups. These LPP results appear to follow closely the behavioral RT data of longer and less accurate emotion categorizations for sad and fearful relative to happy expressions, irrespective of dysphoria status.

## Caveats and Conclusion

The sample of participants was relatively small and consisted of predominantly female undergraduate students: this may limit the generalizability of our findings to other samples (e.g., older individuals, community samples). Also, the possibility that transient depressed mood drives biases in emotional information processing deserves further investigation using mood-induction tasks (Liotti and Mayberg, 2001).

It would be interesting to adopt this covert-overt emotion paradigm in clinically depressed patients. In light of the evidence of impaired attentional disengagement from symptom-congruent (i.e., depression-related) stimuli (Yiend, 2010; Epp et al., 2012) it would be expected to find effects of depression on the LPP to emotional faces in the overt task, similar to other findings showing effects of depression on the P300 to neutral and emotional stimuli.

Both state and trait anxiety (as measured by the STAI) differed significantly among the high and low dysphoria groups. Particularly trait-anxiety was highly correlated to BDI scores, supporting the notion that stress and anxiety may be precipitating factors to develop significant depression (Gotlib, 1984). However, despite some evidence that the BDI-II and STAI may adequately differentiate between anxiety and depression, a number of items and factor analyses have found that the STAI may not adequately measure anxiety as a distinct construct but may be measuring general negative affect and depressive symptoms as well (Hill et al., 2013).

Arousal ratings revealed a small but significant difference between happy and sad expressions. It has been found that N170 is sensitive to perceived arousal of facial expressions rather than categories of emotional expressions. This influence run against a possible mood-congruent bias and may have partially obscured it (Almeida et al., 2017).

Our mass univariate approach, while allowing a more comprehensive analysis of the full time-course of the neural activity over the posterior half of the scalp surface in an unbiased way, does present some trade-offs relative to the traditional ERP approach based on time windows and predefined scalp sites. While it is less sensitive to small effect sizes, it is more able to detect activity from distributed than focused neural sources, and it is relatively insensitive to latency information.

These limitations notwithstanding, to our knowledge, this is the first ERP study to investigate the ERP correlates of the influence of dysphoria on the processing of covert and overt emotional expressions in a within-subject design and to report evidence that dysphoria affects early automatic stages of face processing independent of attentional deployment and emotional category, with a left-lateralized N170 amplitude increase in high dysphoria relative to low dysphoria individuals and an N170 amplitude increase for happy relative to fearful faces unique to the high dysphoria group.

## Data Availability Statement

The original contributions presented in this study are included in the article/Supplementary Material, further inquiries can be directed to the corresponding author.

## Ethics Statement

The studies involving human participants were reviewed and approved by Simon Fraser University Research Ethics Board. The patients/participants provided their written informed consent to participate in this study.

## Author Contributions

FJ-F: study ideation, programming, data collection, first analysis, and manuscript writing and editing. AM: data analysis, figures, and manuscript editing. JG: study ideation. KK: data coordination and analysis. AC: data analysis and figures. PS: data interpretation and manuscript editing. ML: project funding and overall supervision, assistance in study ideation, and manuscript writing. All authors contributed to the article and approved the submitted version.

## Conflict of Interest

The authors declare that the research was conducted in the absence of any commercial or financial relationships that could be construed as a potential conflict of interest.

## Publisher’s Note

All claims expressed in this article are solely those of the authors and do not necessarily represent those of their affiliated organizations, or those of the publisher, the editors and the reviewers. Any product that may be evaluated in this article, or claim that may be made by its manufacturer, is not guaranteed or endorsed by the publisher.
