# Supplementary material for: Spatiotemporal Dynamics of Covert vs. Overt Emotional Face Processing in Dysphoria
Source: Front Behav Neurosci. 2022 Jul 5;16:920989. doi: 10.3389/fnbeh.2022.920989 (PMC9296982; doi:10.3389/fnbeh.2022.920989)
Supplement: Supplementary file 2 [file Image_1.pdf]

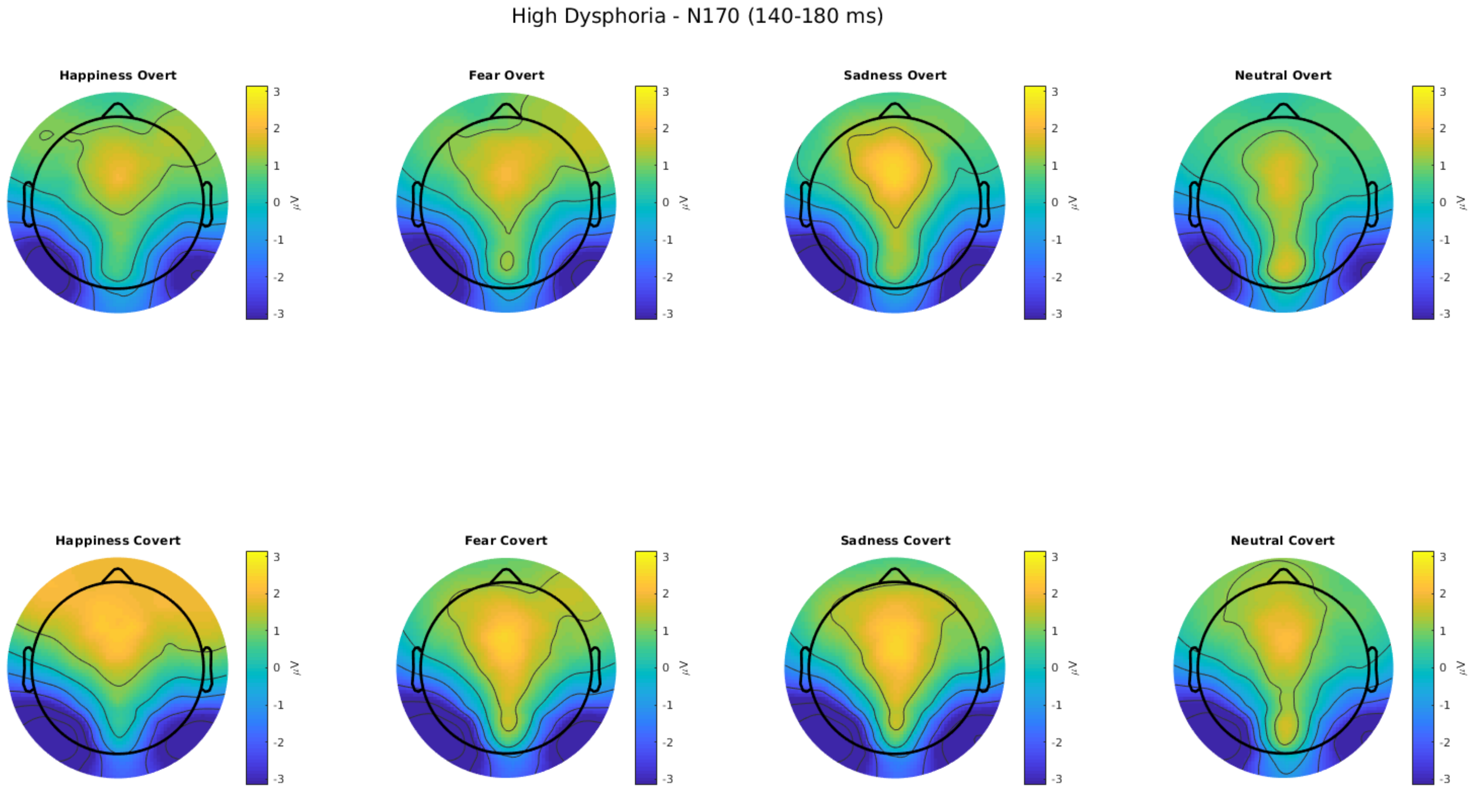

Supplementary Figure 1: Scalp maps showing the N170 amplitude in the High Dysphoria group for each emotional expression and experimental condition
